# A human right to pleasure? Sexuality, autonomy and egalitarian strategies

**DOI:** 10.1136/jme-2023-109011

**Published:** 2023-06-05

**Authors:** Jon Wittrock

**Affiliations:** Global Political Studies, Malmö Universitet, Malmö, Sweden

**Keywords:** Ethics, Human Rights, Philosophy

## Abstract

A growing focus on pleasure in human rights discourse has been used to address patterns of sexual exclusion, often when addressing the problems of people with disabilities (PWD). As convincingly argued by Liberman, however, not all PWD suffer from sexual exclusion, and not all who suffer from sexual exclusion are PWD. Danaher and Liberman have thus argued in various ways for a broader range of measures, addressing sexual exclusion. This article builds on previous research and offers a conceptual framework for addressing sexual pleasure and exclusion in terms of human rights. It argues that human rights aim to safeguard autonomy, which is interpreted as multidimensional. It, thus, divides autonomy into the four dimensions of liberty (freedom from threat and coercion), opportunity (options to choose between), capacity (what an agent is capable of doing) and authenticity (the extent to which choices are genuine). Furthermore, it distinguishes between distinct egalitarian strategies, which offer different problems and possibilities, and may be combined. Thus, there is direct egalitarian distribution, indirect egalitarian distribution, baseline or threshold strategies and general promotion strategies. By way of conclusion, the importance of sexual authenticity as the ultimate aim of sexual rights is emphasised.

## Introduction

‘Sex is not a sandwich’; it cannot be legitimately divided and distributed.^1–3^ Or can it? Several scholars have argued that there are ‘sexual goods’ that we can think of in terms of distribution and egalitarian justice. The key candidate is pleasure, although there also seem to be the associated, often empirically overlapping, but analytically distinct ‘goods of intimacy, romance and love.’^4^

A growing focus on pleasure in human rights discourse has been used to address patterns of sexual exclusion, often when addressing the problems of people with disabilities (PWD). The focus on pleasure is partly polemical: ‘recognising sexual pleasure as a fundamental human right’ may serve as a contrast to ‘conservative approaches that define sex and sexuality solely in reproductive terms.’^5^ Positively, however, it has been used, for example, to argue for changes to sex education, and for providing the information required for filtering ‘unhealthy messages about sexual pleasure…’^6^ However, it has also been used, more controversially, to propose changes pertaining to the regulation of access to sexual opportunities and some worry that ‘formalising this right to sexual pleasure will entrench the competition between women’s human rights and men’s entitlement to use women as sexual objects.’^7^

Pleasure is a complex concept, involving biological, emotional and cognitive aspects: ‘Sexual pleasure has been described as the overall enjoyment derived from sexual interaction […] including a myriad of positive feelings stemming from sexual stimulation…’^8^ The notion that redistributive politics could have something to do with access to sexual opportunities is not new, and neither is the criticism that trying to redistribute access to partners implies reducing the latter to sexual objects.^9^

In order to avoid crude or inconsistent conceptualisations of sexual distribution, this article will provide a philosophical framework for thinking about what human rights should aim to safeguard and distinguish between different egalitarian strategies. Finally, the combination of these conceptual propositions will be used to structure potential public measures, drawing on suggestions from previous research. This article is primarily constructive rather than prescriptive: it aims to offer a roadmap for exploring topics of sexuality and distribution in a shared conceptual space. By way of conclusion, it will be argued, however, that while pleasure is important, our ultimate aim should be to promote sexual authenticity, that is, the ability to make genuine decisions regarding sex.

## Previous research on sexual exclusion

Sexual exclusion may result from mental and/or physical conditions which make desirable sexual relations difficult to attain without social support. Previous research refers to several problems affecting PWD, that is, residing in controlled environments, coercion from parents, caregivers or partners, and a lack of information, time to make decisions, and support from significant others; suggestions for improvement include access to sexual surrogacy and sex doulas.^10^

Appel argues that some PWD should be exempted from prohibitions on prostitution.^11^ To this, Di Nucci retorts that ‘universal positive sexual rights are incompatible with universal negative sexual rights.’^12^ Thus, he argues for a system of volunteers instead. However, the notion of an intrinsic clash between negative and positive rights rests on a failure to grasp that rights differ in weight and specificity, and that there is a crucial analytical difference between rights being universal and rights being absolute. In actual fact, Appel’s proposal would not imply a guaranteed access to sex with other people, implying that the latter would have to be forced. It would only imply that if there are people who voluntarily agree to be sex workers, PWD should have access to the services offered by these people.

Nevertheless, it might be objected that many people advance strong moral arguments against paid sex work.^13^ Furthermore, Appel’s proposal restricts its discussion of consequences of a right to pleasure to PWD. Asa Liberman observes, focusing on ‘disability as a proxy for sexual exclusion’ simultaneously stigmatises PWD and obscures the actual extent of sexual exclusion.^14^ It would thus make more sense to consider a potential right addressing sexual exclusion more broadly.

Along these lines, Danaher argues ‘that having access to meaningful sexual experiences is an important part of the good life, that some people are unjustly sexually excluded, and that we ought to think of this as a problem of distributive justice…’ Danaher, thus, proposes measures ‘removing barriers to meaningful sexual experience (such as legal bans on certain forms of sexual expression) and facilitating positive sexual experiences (through, eg, education or technological aid).’^15^ Relevant mechanisms of exclusion include personal (eg, shyness), social (eg, criminalising homosexuality) or natural (evolved instinct leading people to favour some attributes over others) factors although these categories are not empirically mutually exclusive, but may rather intersect in various ways. Thus, Danaher mentions measures such as a positive right to sex education stressing critical reflection on sexual desires and practices, information about common prejudices (eg, viewing disabled people as asexual), access to sex aid and sex toys, training for health professionals, and assisting people in meeting sexual partners.^15 16^

Liberman points out that there are distinct forms of sexual exclusion, proposing the following three categories: ‘(1) lack of access to sexual pleasure; (2) lack of partnered sex and (3) lack of the social or psychological validation that comes from being seen as a sexual being.’^17^ Pertaining to the first category, Liberman argues that access to solo sexual pleasure should be a positive right and those who require assistance to attain solo sexual pleasure could be helped by trained assistants (which should have a right to deny such assistance, and should never be pressured into providing it) or by mechanical sexual assistance, for example, by ‘sex robots’; the latter should be designed in such a way as to be mindful of the risks of dehumanising or objectifying women. Those who deny themselves solo pleasure due to ignorance, however, should be aided by receiving quality sexual education, which might also be a helpful antidote to ‘a myriad of false and harmful myths about sexual pleasure stemming from pornography, media and popular culture.’^18^

As for the second category, Liberman considers which limits to a negative right to freely choose sexual partners are legitimate (involving issues of incest, power differentials, prostitution, schools, colleges and prisons) and offers a reflection on how to address the fact that some people outside of these contexts will simply suffer from sexual exclusion, simply because they are unable to find willing partners. On the one hand, there are specialty dating apps that might assist people in finding each other; on the other hand, however, there is the deeper issue of reflecting on our own desires. Referring to Srinivasan, Liberman suggests that we think about whether our preferences reflect macrosocial systems of oppression, biases and the kind of media we consume.^19 20^

Concerning the third category, Liberman observes that virginity might be increasingly stigmatising with age, that having had sex is often viewed as a crucial step towards adulthood, and that a narrow focus on what counts as ‘real’ sex excludes can be detrimental to those who do not wish to, or are unable to, engage in heterosexual intercourse. In addressing these tendencies, however, Liberman argues that, while a lack of validation can indeed be painful, the proper response is to attempt to change our norms of validation, to the extent that these ‘constitute a harmful and patriarchal constraint on our flourishing.’^21^ Transcending these constraints might be a prolonged process without any guarantee of success, but starting steps might entail advocating ‘for a broader range of depictions of sexual inexperience in popular culture’ and being mindful ‘about how we are framing discussions about sexual inexperience in our dating app profiles or in conversations with our friends.’^22^

To conclude this section, there is a growing literature reflecting on problems of sexual exclusion and proposing various ways of addressing it by public measures, resting on normative presuppositions of universal, egalitarian rights, both negative and positive. In the following sections, I will offer an analytical framework for structuring debates on these topics in terms of dimensions of autonomy and different egalitarian strategies.

## Human rights and multidimensional autonomy

The Preamble to the Universal Declaration of Human Rights (UDHR) proclaims in its opening sentence that ‘recognition of the inherent dignity and of the equal and inalienable rights of all members of the human family is the foundation of freedom, justice and peace in the world’. One view thus holds that ‘human dignity,’ as Habermas argues, is ‘the moral ‘source’ from which all of the basic rights derive their sustenance.’^23^ Many critics have of course observed, however, that the concept of human dignity in the context of human rights discourse is not exactly clear, although this need not necessarily be viewed as a disadvantage: as Jacques Maritain put it, ‘Yes, we agree about the rights but on condition no one asks us why.’^24^ Nevertheless, many do find this lack of clarity annoying, and argue for swapping dignity for autonomy.^25 26^

In the pursuit of a philosophical basis for human rights, it is possible to work internally or externally, top-down or bottom-up: that is, seeking to inductively abstract normative premises from actual rights, or advocate changes of the rights drawing from our own normative premises.^27^ I will do both: I will propose a normative content, that makes sense given what human rights actually aim to maintain, but that may also, once clarified, be used to argue for changing or adding to existing structures.

In the following, I propose that we do analyse dignity in terms of autonomy. That this is a sensible point of departure is clear from simply reading the UDHR, including its preamble, which repeatedly emphasises freedom and liberty, and cautions against the risks of tyranny and oppression. Working at least partly internally, one has to acknowledge that international human rights, as emanating out of the UN Charter, the UDHR and the International Bill of Human Rights in its entirety, does have their roots in a specific conceptual and philosophical heritage, which incorporates an emphasis on autonomy.

In order to argue credibly for autonomy as a normative basis for human rights, however, one must acknowledge its complex and multidimensional character, implying that different dimensions of it clash with each other. This necessitates difficult decisions and trade-offs, which are the subject of political and public debates as well as decisions by courts and committees.

The notion that human rights protect human autonomy is not new. As Ignatieff formulates one strand of thought, ‘Human rights matter because they help people to help themselves. They protect their agency. By agency, I mean more or less what Isaiah Berlin meant by ‘negative liberty’…’^28^ Here, however, we immediately encounter a paradigmatic debate, arising out of the tension between negative and positive liberty.^29^ Human rights target not simply violations of our negative liberty in the form of illegitimate interventions—violence, torture, arbitrary arrests, etc—but also privations, limiting our positive liberty or capacity for action.

I will use the term liberty to refer to what is often called negative liberty. Positive liberty, for its part, may be further broken down into at least two constituent components: capacities and opportunities. Regardless of our exact stance on the responsibility of public action, it is difficult to deny that opportunities, presenting a range of choices, are crucial for most people’s actual enjoyment of autonomy. Most people, in order to actually enjoy their autonomy, require opportunities to choose between, including others to interact with, and things to explore. Without a world of entities and processes to interact with, there would be ‘nothing to do and nowhere to go, nothing to be and no-one to know’.^30^

This stance, in turn, may result in an emphasis on the development of people’s *capacities* as a core aspect of human dignity. Nussbaum refers to Karl Marx’s Economic and Philosophical Manuscripts as a way of illuminating this intuition: ‘For the starving man, it is not the human form of food that exists, but only its abstract being as food…’^31^ What Marx is implying, Nussbaum argues, is that ‘certain human functions…seem to have a particular centrality in any life one might live’.^32^ There is a fully human and a less than human way of performing these functions, and human rights, Nussbaum argues, should support the development of the former, and protect us against privations resulting in the latter. While drawing on Nussbaum, however, I use a slightly different terminology, distinguishing between capacities (eg, functioning digestive system, absence of allergies and eating disorders) and opportunities (the actual range of food and drink available to choose between). Our capacities may extend the range of our opportunities. For example, if I am able to digest a certain potential source of nutrition, that fact in itself transforms that potential source of nutrition, for me, into an opportunity to eat. Some of the consequences of such variations are minor, whereas others are overwhelming, rare, and potentially life-changing.

Finally, there is what I propose to call the dimension of authenticity, that is, the extent to which an agent’s choices are genuine, in the sense of being based on adequate information, the capacity for critical reflection, and the absence of threat and coercion. The dimension of authenticity thus combines aspects of all preceding dimensions: an authentic choice should not be made under threat or coercion, it should be based on access to accurate information (opportunity) and the capacity for critical reflection. No choice will ever be perfectly authentic, but it is nevertheless difficult to deny that any choice can be more or less so. In my usage, the dimension of authenticity thus relies on criteria of all of the preceding dimensions, and is closely related to Griffin’s notion of normative agency as well as Joseph Raz’s conditions of autonomy.^33 34^

What we have here, I argue, are four dimensions of autonomy, which are all of key relevance when considering human rights. Expanding one of these dimensions, however, may encroach on the others and any specific configuration of compromises between different dimensions may be questioned regarding its spatiotemporal distribution. In other words, critics may argue that expanding autonomy in the present may threaten its status in the future, and expanding or limiting autonomy spatially similarly entails conflicts concerning its distribution. Analysing human dignity in terms of multidimensional autonomy thus indicates difficult trade-offs between its four major constituent dimensions.

## Sexuality and distribution: egalitarian strategies

Even if we accept the idea that those who are unfortunate in terms of access to romantic and sexual partners ought to be aided by way of public measures, it is not necessarily clear what makes someone unfortunate in terms of access to sex: are we to consider, for example, number of partners, or frequency of sexual activities, or perhaps access to, or frequency of, good sex, or is it perhaps access to sex with people we are in love with, or are strongly sexually attracted to, that actually counts?

I believe it would be helpful at this point to distinguish between four different egalitarian strategies:

1. Direct egalitarian distribution strategies directly distribute a good x so that everyone has a relevantly equal share of x. For example, distributing US$40 to four people, everyone would get US$10 each, but we would not care if one person got two US$5 bills and the other one US$10 bill; that would not be a relevant difference.
2. Indirect egalitarian distribution strategies use a direct egalitarian distribution strategy for a good or resource that can be used by people to gain in principle equal access to other goods they themselves select. For example, we might give four pupils US$10 each to purchase the food and drink of their choice during a school trip.
3. Baseline or threshold strategies aim to guarantee that everyone has access to a relevant baseline pertaining to some good. For example, all pupils get at least US$10 each for food and drink, but some get more (eg, by their parents).
4. General promotion strategies: some good x is generally promoted across society as a whole, using a range of strategies. For example, promoting healthy food for school children, governments might subsidise healthy school meals, require schools to offer special classes on nutrition, prohibit ads that increase eating disorders and introduce a tax on sugar.

Not all of these strategies demand a good that can be easily quantified and directly distributed. Obviously, pleasure cannot be directly distributed, but negative rights to refuse sexual engagement are distributed directly and equally; also, informational material may be distributed in this way. We need not restrict ourselves to one strategy, however, but may combine several in order to achieve certain aims.

I have argued for multidimensional autonomy as the overarching aim of human rights. This theoretical framework allows us to situate different proposals for public measures, as potentially complementary, but calling for trade-offs in terms of prioritisations. Drawing on previous research, and combining dimensions of autonomy and egalitarian strategies, we may envisage the following potential public measures relevant to a human right to pleasure, as listed in table 1.

**Table 1:** Dimensions of sexual autonomy and egalitarian strategies

	Direct distribution	Indirect distribution	Baseline strategy	General promotion
Liberty	Equal negative rights to refuse sex and freedom from unwanted control and surveillance			Campaigns against sexual coercion involving information, education and legislative measures
Capacity	Distribution of standardised basic information to all pupils in a certain age group	Right to sexual education, subsidised counselling and partner therapy	Assistance in developing sexual capabilities by sex doulas or sex surrogates	Campaigns promoting shared pleasure, disseminating information widely through public broadcasting
Opportunity		Sex care subsidies for all adults; may be used for counselling, therapy, healthcare consultation and, if legally appropriate and deemed morally acceptable, paid sex work	Subsidising access to mechanical assistance or trained care providers aiding masturbation	Access to realistic portrayals of a broader range of sexual norms and experiences through public broadcasting; subsidising and regulating fair and accessible dating apps

Table 1 visualises something that is easily glossed over: that not only sexual exclusion, but a lack of partnered or solo sexual pleasure, as well as more opaque coercive mechanisms, may be distributed widely across society and may thus not be targeted by more focused measures (baseline strategies). General promotion strategies aim to affect what we can call the general opportunity structure, rather than to provide a baseline of opportunities. Combining several strategies allows for a broader effect, than to only focus on baselines or, for example, changing norms.

Some of the proposals surveyed above would likely be more controversial than others, although the reasons for controversy may vary and may also overlap to various extent pertaining to specific proposals. In some cases (eg, sex care subsidies for all adults), it might be a question of the costs and fears of imposing excessive burdens,^35^ and it would be best to think of potential proposals in terms of extending from minimal, guaranteed measures (eg, equal negative rights to refuse sex) to more ambitious aims, which might be subject to progressive realisation^36^ or optional public measures rather than human rights norms. In other cases, measures might clash with cultural and religious sensitivities. Thus, campaigns promoting shared pleasure and access to realistic portrayals of a broader range of sexual norms and experiences might become controversial for those reasons. In part, there is no way to guarantee the absence of some degree of controversy surrounding any potential public measures (pertaining to sexuality or anything else), but consulting with representatives of religious and cultural communities might be a feasible path to alleviate such concerns. Assistance for the development of sexual capacities and access to aided masturbation might become controversial for all of the reasons indicated above (as might, ultimately any proposal), but also due to the fear of implicit coercion (eg, related to temporary or contract employment) pertaining to the provision of such assistance; this risk must be carefully avoided.

Ultimately, what matters most is not pleasure per se, but that people are supported in making authentic sexual choices. This implies that we, as Srinivasan puts it, ‘interrogate the formation of our desires’.^37^ This, however, does not merely entail questioning who we desire, but also what we desire, that is, frequency and kinds (or abstention from) of sexual activities. Pursuing sexual authenticity requires protecting people from threat, coercion and unwanted surveillance, and providing information as well as training in critical reflection, including reflection on the origins and context of desires, as well as the short-term and long-term consequences of sexual practices and relations. The focus on authenticity is equally important on all sides of the sexual equation; it affects those seeking sexual opportunities, or to develop their sexual capacities, but also those who may provide assistance in these areas.^38^

The framework presented above offers a roadmap for further inquiry, in terms of situating different proposals for potential public measures in a shared conceptual space, tied to an interpretation of human dignity in terms of multidimensional autonomy. All dimensions of autonomy, I have argued, are necessary for the development of a credible conception of human dignity as a desirable aim for human rights, and we should not wholly sacrifice any at the expense of others. However, the extent to which any dimension is supported by public measures will vary as a consequence of legal and political decisions, as well as the availability of resources. Moving forward, a particular emphasis should be put on further understanding sexual authenticity, both empirically and conceptually.

## Data Availability

Data sharing not applicable as no datasets generated and/or analysed for this study.
